# Neurobehavioral outcomes of neonatal asymptomatic congenital cytomegalovirus infection at 12-months

**DOI:** 10.1186/s11689-024-09533-0

**Published:** 2024-04-18

**Authors:** Sally M. Stoyell, Jed T. Elison, Emily Graupmann, Neely C. Miller, Jessica Emerick, Elizabeth Ramey, Kristen Sandness, Mark R. Schleiss, Erin A. Osterholm

**Affiliations:** 1 Institute of Child Development, University of Minnesota, Minneapolis, MN USA; 2 Department of Pediatrics, University of Minnesota, Minneapolis, MN USA; 3 Masonic Institute for the Developing Brain, University of Minnesota, Minneapolis, MN USA

**Keywords:** Congenital CMV infection, Intellectual disability, TORCH infections

## Abstract

**Background:**

Congenital cytomegalovirus (cCMV) is the most common congenital viral infection in the United States. Symptomatic infections can cause severe hearing loss and neurological disability, although ~ 90% of cCMV infections are asymptomatic at birth. Despite its prevalence, the long-term neurobehavioral risks of asymptomatic cCMV infections are not fully understood. The objective of this work was to evaluate for potential long-term neurobehavioral sequelae in infants with asymptomatic cCMV.

**Methods:**

Infants with cCMV were identified from a universal newborn cCMV screening study in a metropolitan area in the midwestern United States. Asymptomatic infants with cCMV were enrolled in a longitudinal neurodevelopmental study (*N* = 29). Age- and sex-matched healthy control infants (*N* = 193) were identified from the Baby Connectome Project (BCP), a longitudinal study of brain and behavioral development. The BCP sample supplemented an additional group of healthy control infants (*N* = 30), recruited from the same participant registry as the BCP specifically for comparison with infants with asymptomatic cCMV. Neurobehavioral assessments and parent questionnaires, including the Mullen Scales of Early Learning, the Repetitive Behavior Scales for Early Childhood (RBS-EC), and the Infant Toddler Social Emotional Assessment (ITSEA) were administered at 12 months of age. Neurobehavioral scores were compared between infants with asymptomatic cCMV and all identified healthy control infants.

**Results:**

Infants with asymptomatic cCMV performed equivalently compared to healthy control infants on the neurobehavioral measures tested at 12 months of age.

**Conclusions:**

These results indicate that at 12 months of age, infants with asymptomatic cCMV are not statistically different from controls in a number of neurobehavioral domains. Although follow-up is ongoing, these observations provide reassurance about neurobehavioral outcomes for infants with asymptomatic cCMV and inform the ongoing discussion around universal screening. Additional follow-up will be necessary to understand the longer-term outcomes of these children.

**Supplementary Information:**

The online version contains supplementary material available at 10.1186/s11689-024-09533-0.

## Background

In the United States, the most common congenital viral infection is congenital cytomegalovirus (cCMV), with an estimated prevalence of 4.5 infections per 1000 live births. In healthy adults, cytomegalovirus typically presents as mild or undetectable. In congenital cases though, symptomatic infections can cause severe disability in the affected infant. Although these sequelae are an important concern in infants and children with symptomatic cCMV, approximately 90% of cCMV infections are asymptomatic at birth [1]. Despite the prevalence of asymptomatic infections, the long-term neurobehavioral risks of asymptomatic cCMV infections are not well understood.

In symptomatic cCMV infections, the most common disease manifestation is sensorineural hearing loss (SNHL) [1, 2]. cCMV is the most common non-genetic cause of SNHL in children, accounting for more than 9% of all cases [3], with the risk of developing progressive SNHL remaining high until 5 years of age [4]. Other common symptoms at birth are petechial rash, jaundice, swelling of the liver and spleen, and neurological abnormalities. Neurological abnormalities can include microcephaly and lethargy in the infant, and upon imaging, 50–70% of symptomatic infants show abnormalities on brain imaging [5]. cCMV is also the most common viral infection in infection-related stillbirths [6]. Later in development, developmental disorders and delays are common, with an estimated 40–60% of symptomatic infants that go on to develop long-term sequelae [7–9]. The pathogenic mechanisms of the impact of cCMV on neurodevelopment are not yet fully understood [10], but animal models suggest cCMV infection interferes with neural stem cells and may induce harmful neuroinflammatory immune processes, with the developmental timing of infection playing a critical role in determining the type and severity of neurodevelopmental impacts [11].

Recent studies of cCMV have shown a relationship with later neurodevelopmental disorders. Retrospective studies of autism spectrum disorders (ASD) using archived dried blood spots have shown cCMV rates above general population prevalence [12]. In general, studies have implicated the immune system in a variety of later neurodevelopmental disorders. Studies of maternal infections, such as CMV, influenza, or toxoplasmosis, demonstrate that these are strong risk factors for a variety of later neurodevelopmental disorders, including ASD, attention-deficit hyperactivity disorder (ADHD), and Tourette syndrome [13, 14]. Animal models suggest that maternal immune activation can lead to a variety of prenatal effects, including neural circuitry modification, CSF flow, and microglial priming, which can trigger a variety of downstream effects [15, 16].

In asymptomatic cCMV, previous studies of the long-term development of asymptomatic cCMV infants have been hindered by the lack of availability of infants to recruit for neurodevelopmental testing who test positive, but do not experience symptoms [9, 17, 18]. Most hospitals only test high risk infants or infants who display symptoms of cCMV, such as hearing loss. Current best estimates suggest ~ 7–11% of asymptomatic infants go on to develop hearing loss, but estimates for cognitive outcomes are less clear [19]. A systematic review of eleven studies reporting cognitive outcomes found no differences between cases and controls at follow-up [19]. These studies, however, had heterogeneous study designs, and many were limited in the neurological and cognitive outcome measures used. Some did not report or account for SNHL at birth, a major potential confounder. Many used gross measures of cognitive impairment, such as the percentage that met a clinical cutoff for impairment based on an IQ or DQ score, which might not detect milder, subthreshold impairments.

With the advent of universal screening programs around the United States and Canada, such as the screening programs that are ongoing in Ontario and Saskatchewan and the program just initiated in Minnesota in 2023 [20], there is a pressing need to understand the longer-term neurobehavioral impacts of asymptomatic cCMV and develop recommendations for best practices surrounding future monitoring of identified asymptomatic infants. As the current literature on longitudinal outcomes of asymptomatic cCMV is limited in size and scope, studies using more precise measures of specific skills are needed to fully understand the neurobehavioral implications for these infants and children. Toward this goal, the study described in this report utilized a prospective longitudinal follow-up of asymptomatic cCMV infants recruited from a universal screening study. The objective of this work was to evaluate for potential long-term neurobehavioral sequelae in infants with asymptomatic cCMV. Multiple neurobehavioral assessments and parent questionnaires, including specific aspects of development and social functioning, were employed in 12-month-old infants in order to evaluate for early signs of neurodevelopmental disability and to measure social-emotional competencies. Results from these evaluations were compared to a modestly large group of healthy control infants.

## Methods

### Participants

**Fig. 1:**
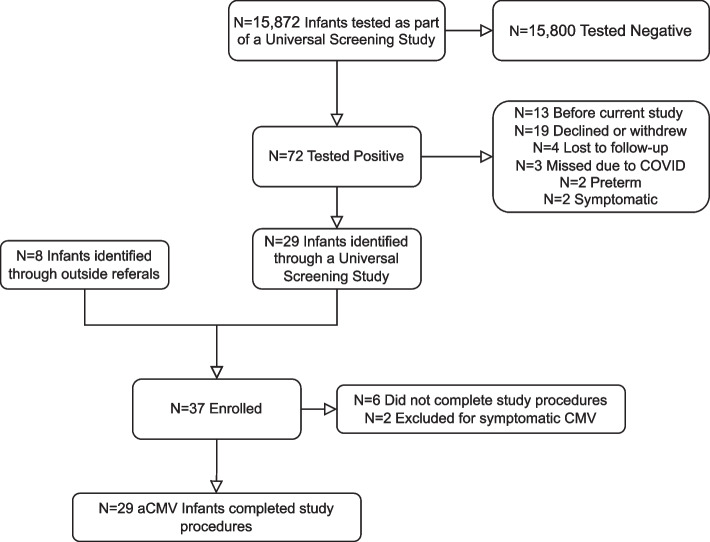
Infants with asymptomatic cCMV were recruited from a universal screening study as well as referrals. In total, 29 infants with asymptomatic cCMV completed study procedures

Infants with cCMV infection were identified from an ongoing universal newborn cCMV screening study in the Minneapolis/St. Paul metropolitan area of the midwestern United States (Fig. 1). All infants born at six hospitals were offered screening at birth for cCMV infection. Screening was conducted via dried blood spot and saliva testing, with positive screens confirmed by a diagnostic urine sample [21]. Infants found to have a symptomatic infection were offered valganciclovir therapy as per standard of care and were excluded from this follow-up study. Infants born less than 35 weeks gestation were also excluded. The remaining asymptomatic infants were eligible for this study. For this study, infants were determined to be symptomatic vs. asymptomatic based on the criteria laid out in a consensus paper on cCMV [22]. All available evaluations were referenced to determine eligibility. The standard of care for evaluation of children with cCMV infection includes several laboratory, neuroimaging, and subspecialty evaluations [22, 23]. The most important component is serial audiological evaluation, since up to 15% of cCMV children (asymptomatic and symptomatic) will have or develop SNHL, which may be delayed in onset. Ophthalmologic evaluation is also recommended, as are laboratory studies including assessment of hepatic enzymes (ALT, AST) and bilirubin. A complete blood count is also recommended, with a particular emphasis on the determination of the absolute neutrophil count and platelet count. Finally, a screening neuroimaging study commonly recommended is cranial ultrasonography, with consideration of a follow-up brain MRI if findings are present on ultrasound examination. Some asymptomatic infants were also identified from referrals outside the hospitals participating in the screening program, such that a portion of the cohort was not recruited as part of a universal screening study. These infants also underwent a comprehensive assessment to determine eligibility, as described above. All asymptomatic infants whose parents consented to participate were then followed longitudinally through the first two years of life, undergoing event-related potential (ERP) assessments, MRI scans, and neurobehavioral assessments at 12 and 24 months old, with the analyses reported in this manuscript focusing on the neurobehavioral assessments at the first timepoint.

Age- and sex-matched healthy control infants were identified from the Baby Connectome Project (BCP), a longitudinal study of brain and behavior of infants 0–5 years that includes MRI scans and neurobehavioral assessments [24]. Infants were eligible for the BCP if they were 1) born at a gestational age of 37–42 weeks, 2) had a birth weight appropriate for gestational age, and 3) had an absence of major pregnancy and delivery complications. Infants were excluded from the BCP if they were born prior to 37 weeks gestational age, had a birth weight lower than 2,000 g, or if they had any major delivery complications. Major delivery complications included neonatal hypoxia or neonatal illness requiring a greater than two-day NICU stay. They were also excluded if they: 1) were adopted, 2) had a first degree relative with autism, intellectual disability, schizophrenia, or bipolar disorder, 3) had any significant medical and/or genetic conditions affecting growth, development, or cognition, or 4) had any contraindication to MRI. Additional exclusion criteria included major pre- and/or perinatal issues including: 1) maternal pre-eclampsia, placental abruption, maternal HIV status, and maternal alcohol or illicit drug use during pregnancy. Finally, infants were excluded from the study if their caregivers were unable to communicate in English at the level required to provide informed consent. Infants were recruited from a research participant registry drawn from statewide Minnesota birth records.

To function as comparators for the ERP assessments, which the BCP did not include, additional healthy control infants were recruited from the same registry as the BCP participants, with the same inclusion criteria as the asymptomatic cCMV infants (> 35 weeks gestational age). Infants were also excluded if their caregivers were unable to communicate in English at a level to provide informed consent. The healthy control participants in this study underwent the same protocol (sans MRI) as the infants with asymptomatic cCMV.

The BCP control group included exclusion criteria above and beyond that of the current study. Of the asymptomatic cCMV and study control participants who had this demographic information available, no participants were adopted, none had major pre/perinatal issues, and only one participant in the control group had a first degree relative with a disorder that would have excluded them from the BCP.

As infants in both control groups were not screened for cCMV, these control groups represent “low likelihood cCMV” groups rather than known negative control groups. It is possible a small number of infants in the control groups would have tested positive for cCMV had they been tested at birth. Given the known prevalence of cCMV in Minnesota of 1:200 newborns [21, 25], this is unlikely to be a substantial number.

All study procedures were approved by the UMN Institutional Review Board.

### Study procedures

#### Developmental assessments

All behavioral assessments were completed at the Center for Neurobehavioral Development (CNBD) at the University of Minnesota. The Mullen Scales of Early Learning [26] was administered using standardized protocols. The Mullen Scales of Early Learning is a standardized neurodevelopmental assessment that measures expressive and receptive language skills, fine and gross motor skills, and visual perception skills. Four of these subscales (expressive language, receptive language, fine motor skills, and visual perception) are combined to derive a composite score, which reflects overall developmental level. For behavioral assessments, hearing aids were used as necessary to accommodate hearing impairment in *N* = 1 infant. Parents also completed two questionnaires about their child’s behavior at 12 months: the Repetitive Behavior Scales for Early Childhood [27] (RBS-EC), and the Infant Toddler Social Emotional Assessment [28] (ITSEA).

The RBS-EC is a parent report questionnaire that captures a range of behaviors in the domains of restrictive and repetitive behaviors. It includes four subscales: repetitive motor, ritual and routine, restricted behaviors, and self-directed behaviors, that all contribute to an overall composite scale. Higher scores indicate higher endorsement or higher frequency of these behaviors. It has been shown that infants who go on to develop ASD show significantly more restrictive and repetitive behaviors by 12 months of age [29, 30]. Repetitive motor movements and repetitive use of objects are also associated with developmental delay in young children [31]. The RBS-EC has been shown to have internal validation across our age range in this study [32] and the longitudinal trajectory across infancy to young childhood has been mapped [33].

The ITSEA is a parent report questionnaire that assesses social-emotional and behavioral development and is designed to identify early deficits or delays. It is comprised of four domains of functioning: externalizing behaviors (e.g., impulsivity, aggression), internalizing behaviors (e.g., withdrawal, anxiety, separation distress, inhibition to novelty), dysregulation (e.g., sleep/eating dysregulation, negative emotionality, sensory sensitivity), and social competencies (e.g., compliance, attention, imitation, empathy, mastery motivation). It also assesses more serious problems in the domains of maladaptive behaviors, social relatedness, and atypical behaviors.

Cronbach’s alpha was greater than 0.85 for both the asymptomatic cCMV and control groups, for both the RBS-EC and the ITSEA, suggesting high reliability across the questionnaires.

#### Analytic strategy

Neurobehavioral assessment scores were compared between infants with asymptomatic cCMV and all identified healthy control infants. All analyses were performed in R version 4.2.1. T-tests, chi-squared tests, and Fisher’s exact test were used as appropriate to analyze differences in demographic variables, including age, sex, race, and ethnicity. Linear regression models including age as a covariate were used to compare neurobehavioral assessment scores between groups. Sensitivity analyses were performed excluding asymptomatic cCMV infants with mild symptoms but who weren’t classified as fully symptomatic.

Data collection for this study was ongoing during the 2020 Covid-19 shut-down, and so multiple visits were delayed. This resulted in a slightly older age for asymptomatic cCMV participants as compared to healthy control participants, especially on the in-person Mullen assessment. To account for this, the BCP participants with Mullen data identified were also slightly older on average. To account for these issues, age was added as a covariate in all models, and sensitivity analyses were run with each of the control groups (BCP and non-BCP) separately.

## Results

### Participants

At the 12 month timepoint, 29 infants with asymptomatic cCMV and 30 control infants participated in neurobehavioral assessments and 193 age-matched infants with the same neurobehavioral assessments were identified from the BCP. Demographic characteristics for each group are reported in Table 1. As household income and parental education data was not collected from asymptomatic cCMV or study control families at data collection visits, this information was collected retrospectively. Additionally, gestational age and birth weight were collected retrospectively from study control participants, as this information was only collected for asymptomatic cCMV and BCP control participants at data collection visits. As noted in the methods, study control participants were younger on average than asymptomatic cCMV participants. Additionally, more BCP participants fell into a lower income bracket, suggesting a wider variability in household resources. Asymptomatic cCMV participants also had a slightly lower gestational age, likely due to the minor difference in inclusion criteria (35 vs. 36 weeks). Of the 29 asymptomatic cCMV participants, 23 were completely asymptomatic, while 6 displayed mild symptoms at birth that were not sufficient to classify them as fully symptomatic cCMV infections, based on criteria from a consensus recommendation [22]. These mild symptoms included SNHL (*N* = 3), intrauterine growth restriction (*N* = 1), or transient thrombocytopenia (*N* = 2). 6 participants were treated with valganciclovir as part of their clinical course. Of those 6, 4 had mild symptoms and 2 were asymptomatic. No asymptomatic cCMV participants in our cohort developed later onset progressive SNHL, although one participant had transient conductive hearing loss (not typically associated with a cCMV infection).

**Table 1:** Demographic Characteristics

	**acCMV (*N* = 29)**	**Study Healthy Controls (*N* = 30)**	**BCP Healthy Controls (*N* = 193)**	***p*-value**
	**mean (sd) or # (%)**	**mean (sd) or # (%)**	**mean (sd) or # (%)**	
Age (months)	13.4 (1.7)	12.1 (0.4)	13.1 (1.4)	< 0.01
Sex (male)	15 (52%)	16 (53%)	95 (49%)	0.91
Race				0.43
White	24 (83%)	26 (87%)	162 (84%)	
Other/More than one race	4 (14%)	4 (13%)	31 (16%)	
Not Reported	1 (3%)	0 (0%)	0 (0%)	
Ethnicity				0.23
Hispanic	0 (0%)	3 (%)	11 (6%)	
Non-Hispanic	27 (93%)	27 (93%)	180 (93%)	
Not Reported	2 (7%)	0 (0%)	2 (1%)	
Maternal Education				0.81
Less than college degree	0 (0%)	0 (0%)	18 (9%)	
College degree	6 (21%)	11 (37%)	81 (42%)	
Graduate degree	9 (31%)	13 (43%)	92 (48%)	
Not Reported	14 (48%)	6 (20%)	2 (1%)	
Household Income				0.02
< 50 k	0 (0%)	1 (3%)	10 (5%)	
50-100 k	1 (3%)	5 (17%)	77 (40%)	
> 100 k	14 (48%)	18 (60%)	104 (54%)	
Not Reported	14 (48%)	6 (20%)	2 (1%)	
Gestational Age (weeks)^a^	38.8 (1.6)	39.7 (1.3)	39.8 (1.0)	< 0.01
Birth Weight (lbs)^a^	7.2 (1.7)	7.7 (1.4)	7.7 (1.0)	0.10
Mild Symptoms^b^	6 (21%)	n/a	n/a	
SNHL at Birth	3 (10%)	n/a	n/a	
Treated with Valganciclovir	6 (21%)	n/a	n/a	

*acCMV* Asymptomatic cCMV, *SNHL* Sensorineural hearing loss

^a^*N* = 5 acCMV, *N* = 4 HC Not reported

^b^See methods section for details on these mild symptoms

### Mullen

**Fig. 2:**
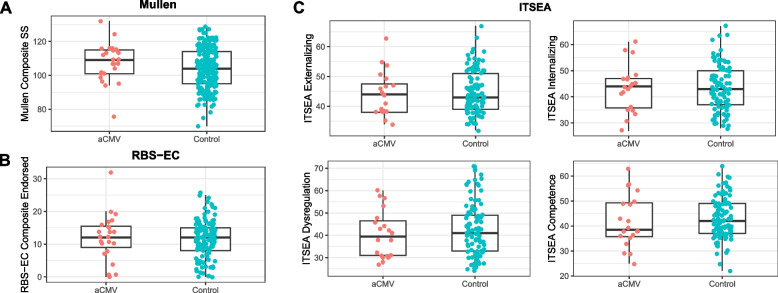
Infants with asymptomatic cCMV performed equivalently to or better than healthy control infants on all of the neurobehavioral measures tested. Pictured here are the Mullen and RBS-EC Composite Scores and the four main ITSEA subscales. On the Mullen composite score, asymptomatic cCMV infants performed better than healthy control infants, *F*(2, 237) = 11.42, *p* = 0.03. On the RBS-EC Composite Endorsed, asymptomatic cCMV infants performed no different than healthy controls, *F*(2,145) = 0.4597, *p* = 0.64. On the ITSEA subscales, asymptomatic cCMV infants also performed no different than healthy controls, with all *p* > 0.4

Infants with asymptomatic cCMV performed equivalently or better than healthy controls on the Mullen Scale and all of its subscales (Fig. 2A Table 2). Overall, on the composite score, asymptomatic cCMV infants performed better than healthy control infants, with a mean of 107.72 compared to a mean of 103.68 for healthy control infants, *F*(2, 237) = 11.42, *p* = 0.03, both just above the population average of 100.

**Table 2:** Mullen Scores by group

	**acCMV (*N* = 25)**		**HC (*N* = 215)**			
	**Mean**	**sd**	**Mean**	**sd**	**B**	***p*-value**
Composite	107.72	11.16	103.68	11.82	-5.40	0.03
Expressive Language	53.28	9.34	50.64	9.47	-3.08	0.13
Receptive Language	46.40	5.74	46.73	8.58	-0.29	0.87
Fine Motor	57.32	8.33	58.07	9.65	0.14	0.95
Gross Motor	53.20	11.18	48.25	10.91	-4.49	0.05
Visual Reception	58.40	9.43	51.71	9.35	-7.75	0.00

The composite subscale is standardized to a mean of 100 with a standard deviation of 15. All other subscales are standardized to a mean of 50 with a standard deviation of 10

*acCMV* Asymptomatic cCMV, *sd* Standard deviation, *B* Beta coefficient

### ITSEA and RBS-EC

No significant differences were found between infants with asymptomatic cCMV and healthy control infants on the RBS-EC composite measures or any of the subscales of the ITSEA or the RBS-EC (Fig. 2B-C, Tables 3 and 4). On the RBS-EC composite score, asymptomatic cCMV infants had a mean of 12.2 items endorsed, while healthy control infants had a mean of 11.7 items endorsed, *F*(2, 145) = 0.46, *p* = 0.64.

**Table 3:** RBS-EC Scores by group

	**acCMV (*N* = 23)**		**HC (*N* = 125)**			
	**Mean**	**sd**	**Mean**	**sd**	**B**	***p*-value**
Composite Endorsed	12.17	7.10	11.65	5.78	-0.63	0.64
Composite Mean Frequency	1.00	0.58	0.89	0.51	-0.12	0.31
Repetitive Behavior Endorsed	7.00	3.06	6.74	2.98	-0.32	0.64
Repetitive Behavior Mean Frequency	2.69	1.32	2.40	1.38	-0.31	0.32
Ritual Behavior Endorsed	1.09	1.95	0.96	1.15	-0.13	0.66
Ritual Behavior Mean Frequency	0.17	0.37	0.13	0.17	-0.04	0.41
Restrictive Behavior Endorsed	2.30	2.27	2.34	1.89	0.03	0.95
Restrictive Behavior Mean Frequency	0.58	0.76	0.60	0.57	0.02	0.91
Self-Injurious Behavior Endorsed	1.78	2.02	1.60	1.77	-0.21	0.61
Self-Injurious Behavior Mean Frequency	0.51	0.78	0.36	0.48	-0.15	0.21

*acCMV* Asymptomatic cCMV, *sd* Standard deviation, *B* Beta coefficient

**Table 4:** ITSEA Scores by group

	**acCMV (*N* = 20)**		**HC (*N* = 101)**			
	**Mean**	**sd**	**Mean**	**sd**	**B**	***p*-value**
Externalizing	44.20	7.52	44.83	7.19	0.48	0.78
Internalizing	43.30	8.91	43.33	8.61	-0.20	0.92
Dysregulation	40.40	10.60	42.42	12.20	1.80	0.54
Competence	41.95	10.40	42.77	8.10	0.76	0.72
Maladaptive	0.06	0.08	0.06	0.09	0.00	0.82
Social Relatedness	1.72	0.27	1.68	0.21	-0.04	0.47
Atypical	0.24	0.17	0.26	0.20	0.02	0.66

*acCMV* Asymptomatic cCMV, *sd* standard deviation, *B* Beta coefficient

### Sensitivity analyses

A sensitivity analysis was performed including only the infants who were completely asymptomatic (*n* = 23) [22]. As with the full analysis, infants with asymptomatic cCMV showed no differences or higher scores on the Mullen composite score and subscores and no differences on the ITSEA or RBS-EC (Supplemental Figure S1). Of the participants excluded in this analysis, one was the only asymptomatic cCMV infant who fell “Below Average” on the Mullen composite score, and another had the highest (> 30) RBS-EC composite endorsed scale. These results did not change when also excluding the two participants who were completely asymptomatic but who were still treated with valganciclovir.

To determine if age differences, or delays caused by the Covid-19 pandemic, between the control groups impacted the results, two more sensitivity analyses were performed: 1) including only the healthy control infants from the BCP, and 2) including only the healthy control infants recruited directly for this study. Including healthy control infants only from the BCP, the results were consistent with the full analysis (Supplemental Figure S2). Including only the healthy control infants recruited directly for this study, there were no differences between groups on any of the measures tested (Supplemental Figure S3).

## Discussion

These results indicate that at 12 months of age, infants with asymptomatic cCMV perform no worse than comparison groups in a number of behavioral domains, including neurodevelopmental skills, restrictive and repetitive behaviors, and social-emotional competencies. This was true across all infants included in this study and in a sensitivity analysis including only fully asymptomatic infants and across two different groups of healthy control infants. These results align with other studies that found no differences between asymptomatic cCMV and healthy control children using putatively less sensitive measures of behavioral and cognitive function [19, 34].

With the onset of universal screening programs, like the recent one in Minnesota, it becomes critical to understand the trajectory of outcomes for asymptomatic cCMV infants, in order to provide guidance to parents and to enable appropriate screenings and interventions throughout infancy and childhood. While not yet observed in this cohort, progressive hearing loss is a common finding, even in infants asymptomatic at birth. With this knowledge, hearing tests are recommended in the months following a cCMV diagnosis [35]. While the findings from this study on neurobehavioral outcomes are tentatively reassuring, there is still much to be learned before establishing guidelines for primary care providers. That said, these results provide a starting point for guidance, even with the knowledge that more work is necessary.

## Limitations and conclusions

This study has some notable strengths. Most of the participants were recruited through a universal screening study that encompassed the Twin Cities metro area. Other research studies have been limited by screening algorithms and guidelines in hospitals that only test infants at high risk for cCMV. By virtue of a universal screening study that tested every consented infant born in the hospital, this study was able to recruit any infant that screened positive and had confirmed cCMV, even if they had no apparent risk factors for adverse neurodevelopmental outcomes, reducing the potential for bias in ascribing adverse sequelae to cCMV infection. Additionally, this study was able to utilize a moderately sized sample of healthy control infants who had participated in the BCP to serve as controls. While the healthy control infants were not screened for cCMV, and thus are a “low-likelihood” group rather than a known negative group, the known prevalence of 1:200 of cCMV in Minnesota suggests that chances of having any substantial number of cCMV infections in the control group are extraordinarily small. Based on this known prevalence, we might have expected at most 1–2 control infants with cCMV.

A corresponding limitation to the universal screening design is that only ~ 56% of the *n* = 52 identified infants who were contacted responded to our invitation to participate, potentially reflecting ascertainment bias. We do not know whether specific factors account for the decision to participate or not, perhaps biasing the sample of asymptomatic cCMV infants. We do know that this group showed a higher Mullen Early Learning Composite score, average ELC = 108, than would be expected in normative epidemiological sample. This may reflect a group with sufficient resources to commit to a year-long longitudinal study of brain and behavioral development, supported by the higher incomes seen among the asymptomatic cCMV participants who provided income data.

Additional limitations warrant mention. The COVID-19 pandemic disrupted data collection. This pushed the timing of data collection later for many infants, most of them asymptomatic cCMV infants. This led to an age difference in the asymptomatic cCMV participants and the healthy control participants, which prompted the inclusion of the BCP healthy controls, who were able to be age-matched to the asymptomatic cCMV participants. This would be more concerning if the asymptomatic cCMV group was statistically younger than the comparison groups and if differences emerged, as differences could be attributable to developmental level. Fortunately, this was not the case. 

This study also included a limited set of observable behaviors and psychological constructs. These cover a variety of domains, including language, motor skills, social-emotional competencies, and restrictive and repetitive behaviors, but of course could not cover all developmental domains. Thus, we can only comment on the behavioral measures specifically tested in this study. These were selected to be more specific than the gross measures of developmental delay typically characterized in this population and targeted toward hypothesized domains of risk.

This analysis was limited to one 12 month assessment. It is possible that certain behaviors that will demonstrate group differences are not yet measurable at this age, or that behavioral differences might arise as cognitive demands increase with age. These infants are being followed out to 24 months and beyond to continue assessing these behaviors. Even at the 12 month visit, we employ targeted measures such as the RBS-EC, which was designed to characterize behaviors associated with developmental delay. By 12 months of age, infants who will go on to be diagnosed with ASD already show group differences in repetitive behaviors [29]. Other neurodevelopmental disorders show similar patterns of early predictions. Infants at high risk for ADHD show group differences by 12 months of age on parent report measures of infant behavior [36]. Therefore, even at 12 months, it noteworthy to see no group differences.

Finally, a limitation of working with the asymptomatic cCMV population is that the definition of a “symptomatic” cCMV infection is still up for debate. This study utilized the guidelines put forth by a group of experts in Rawlinson et al. in 2017, but these categorizations still differ across other expert groups [22]. Additionally, due to inconsistencies in these definitions and differences in standard clinical care across hospitals and providers, some infants with asymptomatic or mildly symptomatic cCMV were treated with valganciclovir therapy, adding additional variability to the clinical course of these infants. A sensitivity analysis excluding those defined as asymptomatic cCMV for this study but who still had mild symptoms also showed equivalent results to the full group of asymptomatic cCMV participants. Future work will determine if these encouraging behavioral results continue through 24 months of age and older, or if subtle deficits present later in infancy and early childhood. As children develop, higher-level cognitive skills emerge, and subtle deficits might start to appear. As children continue into school, with higher academic demands placed on them, if any deficits do exist, they may start to become even more evident. Additionally, known signs of cCMV infection can appear even later into childhood, most notably progressive SNHL. Longer-term follow-up of asymptomatic cCMV participants will be needed to confirm that such infections truly remain asymptomatic over time, or if there are other neurobehavioral differences that may eventually become manifest.

In conclusion, initial evidence from a longitudinal universal screening study in infants with asymptomatic cCMV showed no evidence for neurobehavioral testing abnormalities at 12 months. Future work will also examine the underlying brain development and physiology, as MRI and EEG scans were also collected in this cohort of infants. Results from those analyses will help determine if there are any anatomical or physiological differences in the brain between asymptomatic cCMV infants and healthy controls. Even though current analyses found no behavioral differences between asymptomatic cCMV and control infants, it is possible that differences in brain development or physiology could predict later onset behavioral differences. Alternatively, if brain development were found to be different, but behavioral measures continued to show no deficits in the asymptomatic cCMV infants, it might reflect a degree of plasticity and reorganization of the brain in response to the cCMV infection that functioned to the extent required to protect asymptomatic cCMV infants against the development of any behavioral differences. Follow-up data will provide further insight into these possible developmental trajectories.

### Supplementary Information

**Additional file 1: Figure S1.** On a sensitivity analysis excluding infants (*N* = 6) with mild symptoms, consistent with the full analyses, asymptomatic cCMV infants again performed equivalently or better on all measures. Of the participants excluded in this analysis, one was the only asymptomatic cCMV infant who fell “Below Average” on the Mullen composite score, and another had the highest (> 30) RBS-EC composite endorsed scale.

**Additional file 2: Figure S2.** On a sensitivity analysis excluding the healthy control group not from the BCP (excluding *N* = 30), the results were consistent with the full analyses, such that asymptomatic cCMV infants again performed equivalently or better on all measures tested.

**Additional file 3: Figure S3.** On a sensitivity analysis excluding the healthy control group from the BCP (excluding *N* = 193), asymptomatic cCMV infants showed no differences with healthy control infants on any of the measures tested.

**Additional file 4: Table S1.** Mullen Scores by group. **Table S2.** RBS-EC Scores by group. **Table S3.** ITSEA Scores by group

## Data Availability

The data used during the current study are available from the corresponding author on reasonable request.

## Abbreviations

cCMV: Congenital cytomegalovirus
ADHD: Attention Deficit Hyperactivity Disorder
ASD: Autism Spectrum Disorder
RBS-EC: Repetitive Behavior Scales for Early Childhood
ITSEA: Infant Toddler Social Emotional Assessment (ITSEA)
BCP: Baby Connectome Project
SNHL: Sensorineural hearing loss
