# Supplementary figures and images for: Neurobehavioral outcomes of neonatal asymptomatic congenital cytomegalovirus infection at 12-months
Source: J Neurodev Disord. 2024 Apr 18;16:19. doi: 10.1186/s11689-024-09533-0 (PMC11025208; doi:10.1186/s11689-024-09533-0)

**A****Mullen**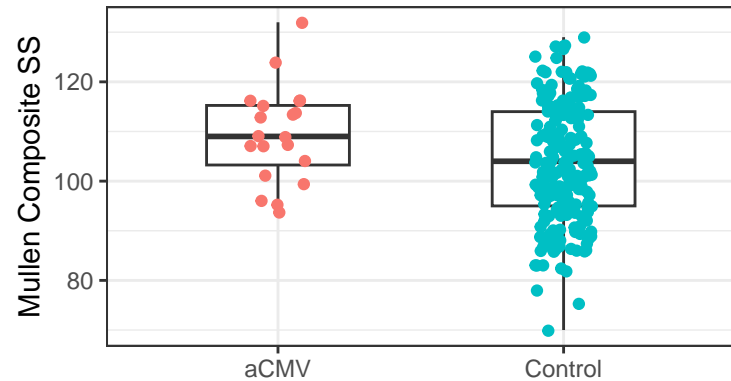**B****RBS-EC**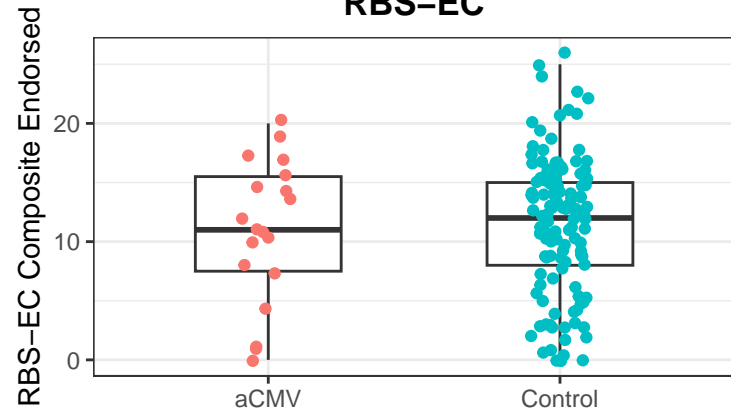**C****ITSEA**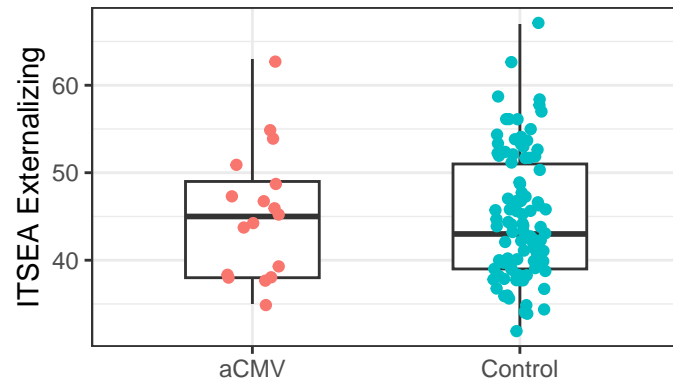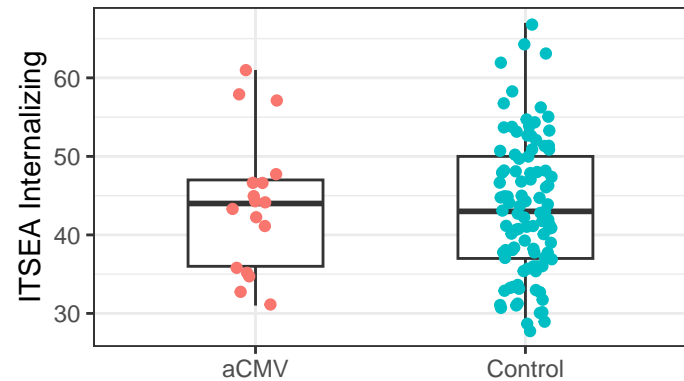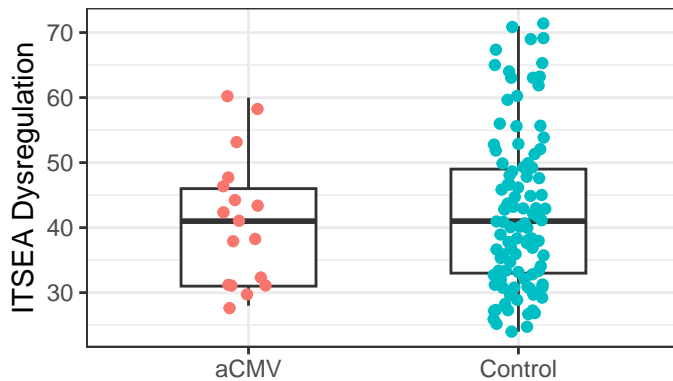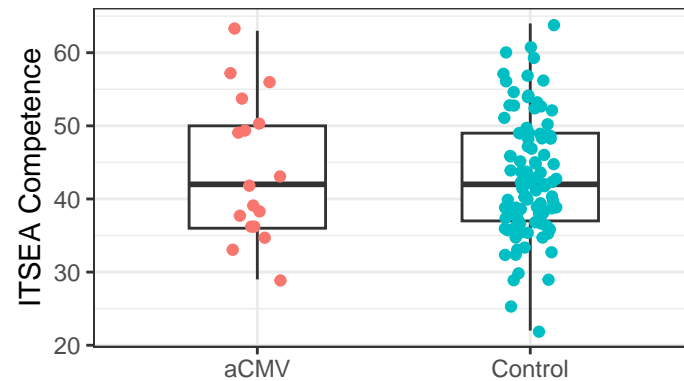

Supplement: Supplementary file 1 — Additional file 1: Figure S1. On a sensitivity analysis excluding infants (N = 6) with mild symptoms, consistent with the full analyses, asymptomatic cCMV infants again performed equivalently or better on all measures. Of the participants excluded in this analysis, one was the only asymptomatic cCMV infant who fell “Below Average” on the Mullen composite score, and another had the highest (> 30) RBS-EC composite endorsed scale. [file 11689_2024_9533_MOESM1_ESM.pdf]

**A****Mullen**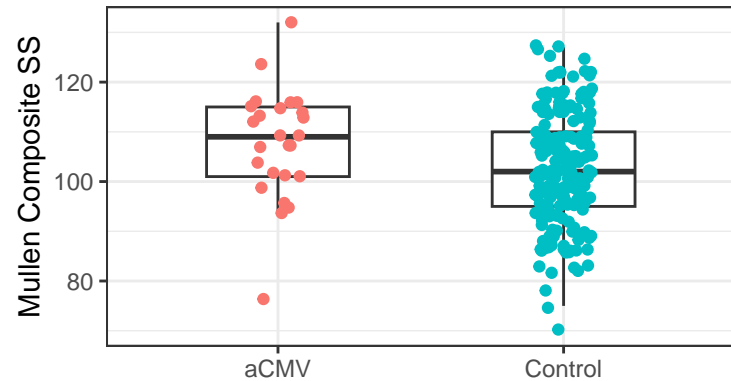**B****RBS-EC**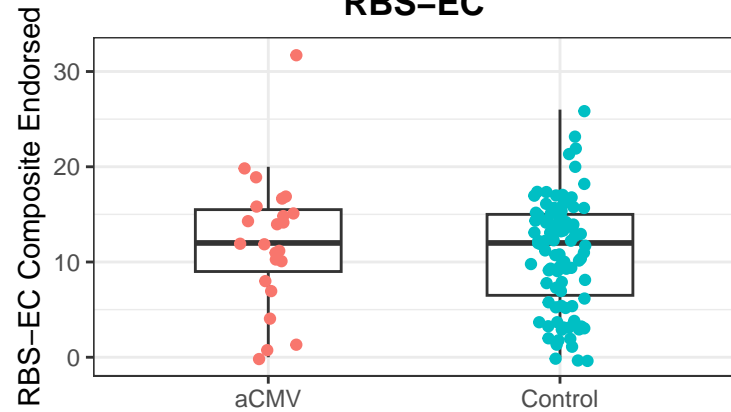**C****ITSEA**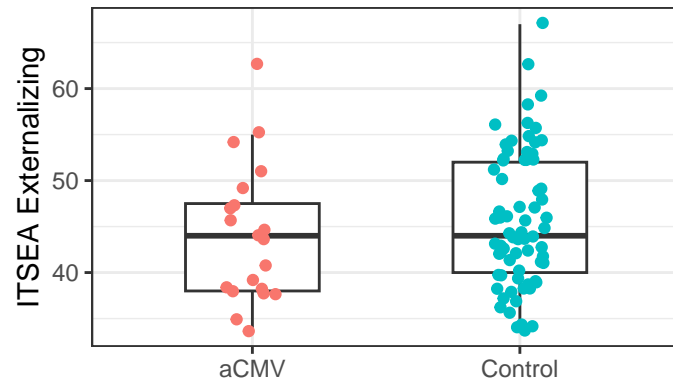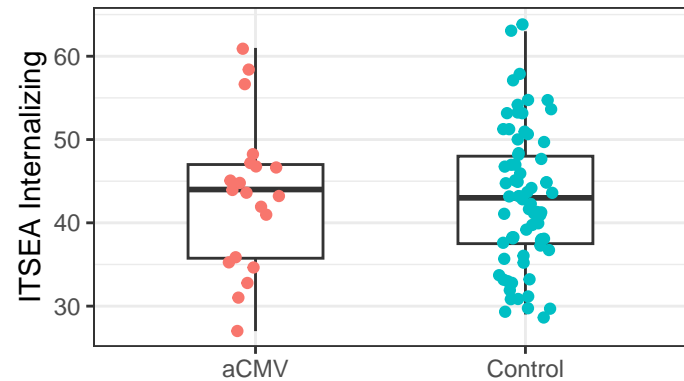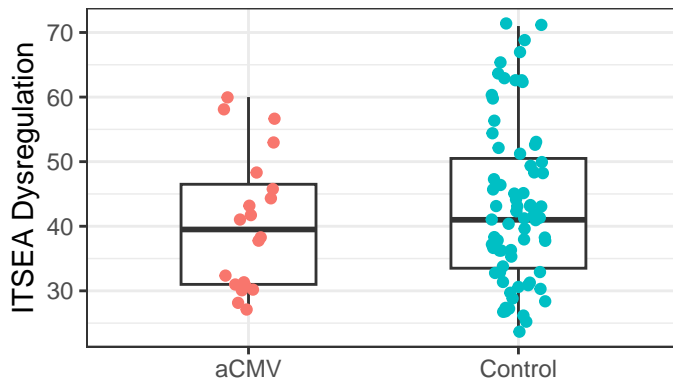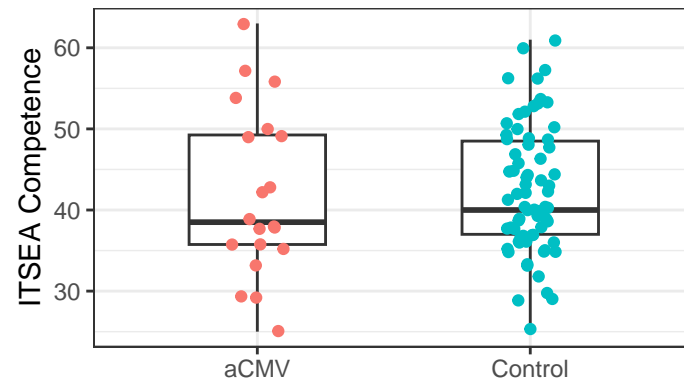

Supplement: Supplementary file 2 — Additional file 2: Figure S2. On a sensitivity analysis excluding the healthy control group not from the BCP (excluding N = 30), the results were consistent with the full analyses, such that asymptomatic cCMV infants again performed equivalently or better on all measures tested. [file 11689_2024_9533_MOESM2_ESM.pdf]

**A****Mullen**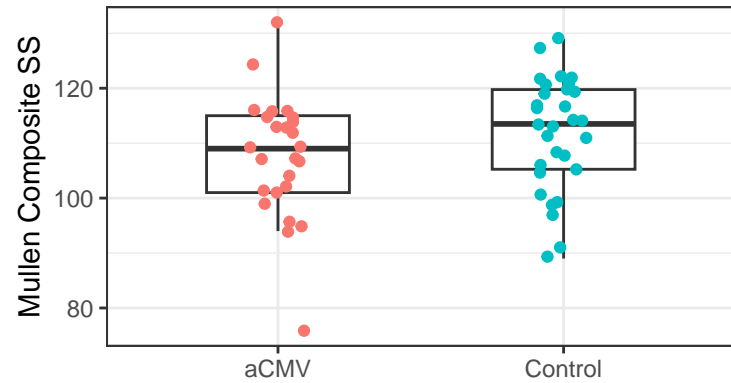**B****RBS-EC**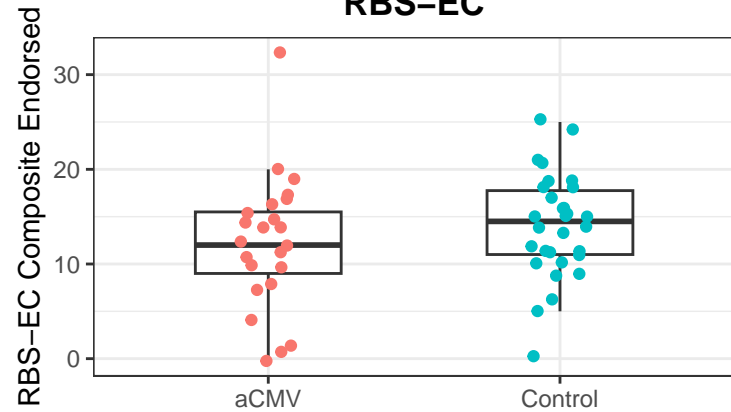**C****ITSEA**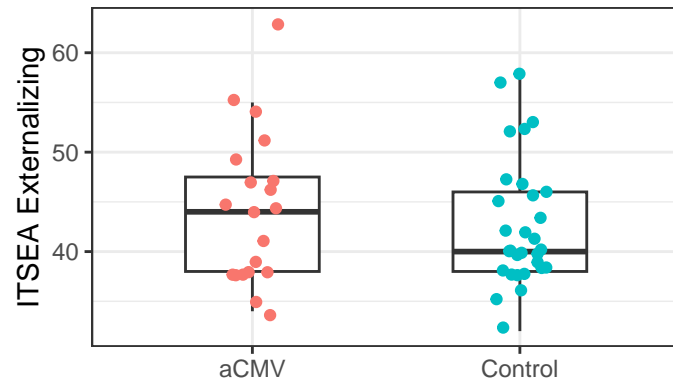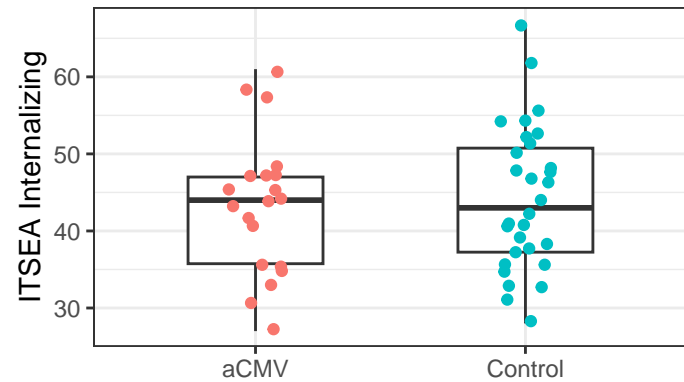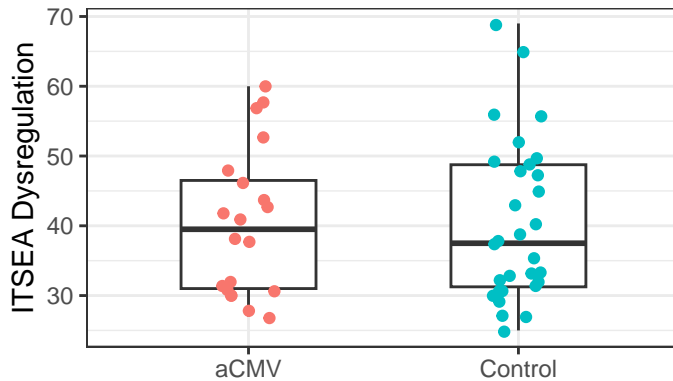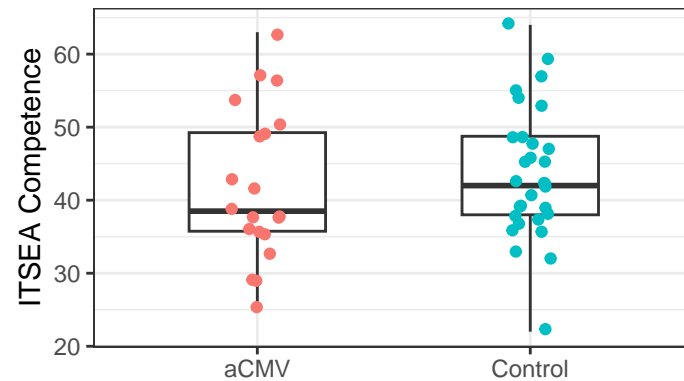

Supplement: Supplementary file 3 — Additional file 3: Figure S3. On a sensitivity analysis excluding the healthy control group from the BCP (excluding N = 193), asymptomatic cCMV infants showed no differences with healthy control infants on any of the measures tested. [file 11689_2024_9533_MOESM3_ESM.pdf]
