# Supplementary material for: Neurobehavioral outcomes of neonatal asymptomatic congenital cytomegalovirus infection at 12-months
Source: J Neurodev Disord. 2024 Apr 18;16:19. doi: 10.1186/s11689-024-09533-0 (PMC11025208; doi:10.1186/s11689-024-09533-0)
Supplement: Supplementary file 4 — Additional file 4: Table S1. Mullen Scores by group. Table S2. RBS-EC Scores by group. Table S3. ITSEA Scores by group [file 11689_2024_9533_MOESM4_ESM.docx]

**Supplemental Tables**

**Table S1.** Mullen Scores by group

|  | **acCMV (N=25)** | | **BCP-HC (N=185)** | | **HC (N=30)** | |
| --- | --- | --- | --- | --- | --- | --- |
|  | **Mean** | **sd** | **Mean** | **sd** | **Mean** | **sd** |
| Composite | 107.72 | 11.16 | 102.36 | 11.54 | 111.87 | 10.23 |
| Expressive Language | 53.28 | 9.34 | 49.66 | 9.29 | 56.70 | 8.38 |
| Receptive Language | 46.40 | 5.74 | 46.43 | 8.85 | 48.57 | 6.44 |
| Fine Motor | 57.32 | 8.33 | 57.58 | 9.78 | 61.03 | 8.34 |
| Gross Motor | 53.20 | 11.18 | 48.49 | 11.20 | 46.80 | 9.00 |
| Visual Reception | 58.40 | 9.43 | 50.83 | 9.38 | 57.13 | 7.22 |

acCMV= Asymptomatic cCMV

The composite subscale is standardized to a mean of 100 with a standard deviation of 15. All other subscales are standardized to a mean of 50 with a standard deviation of 10.

sd=standard deviation; B=beta coefficient

**Table S2.** RBS-EC Scores by group

|  | **acCMV (N=23)** | | **BCP-HC (N=95)** | | **HC (N=30)** | |
| --- | --- | --- | --- | --- | --- | --- |
|  | **Mean** | **sd** | **Mean** | **sd** | **Mean** | **sd** |
| Composite Endorsed | 12.17 | 7.10 | 10.91 | 5.68 | 14.00 | 5.53 |
| Composite Mean Frequency | 1.00 | 0.58 | 0.83 | 0.51 | 1.08 | 0.48 |
| Repetitive Behavior Endorsed | 7.00 | 3.06 | 6.39 | 3.12 | 7.87 | 2.16 |
| Repetitive Behavior Mean Frequency | 2.69 | 1.32 | 2.23 | 1.42 | 2.94 | 1.13 |
| Ritual Behavior Endorsed | 1.09 | 1.95 | 0.86 | 1.15 | 1.27 | 1.11 |
| Ritual Behavior Mean Frequency | 0.17 | 0.37 | 0.13 | 0.18 | 0.16 | 0.14 |
| Restrictive Behavior Endorsed | 2.30 | 2.27 | 2.17 | 1.80 | 2.90 | 2.11 |
| Restrictive Behavior Mean Frequency | 0.58 | 0.76 | 0.56 | 0.54 | 0.72 | 0.65 |
| Self-Injurious Behavior Endorsed | 1.78 | 2.02 | 1.48 | 1.73 | 1.97 | 1.88 |
| Self-Injurious Behavior Mean Frequency | 0.51 | 0.78 | 0.33 | 0.47 | 0.45 | 0.49 |

acCMV= Asymptomatic cCMV

sd=standard deviation; B=beta coefficient

**Table S3.** ITSEA Scores by group

|  | **acCMV (N=20)** | | **BCP HC (N=71)** | | **HC (N=30)** | |
| --- | --- | --- | --- | --- | --- | --- |
|  | **Mean** | **sd** | **Mean** | **sd** | **Mean** | **sd** |
| Externalizing | 44.20 | 7.52 | 45.73 | 7.35 | 42.70 | 6.42 |
| Internalizing | 43.30 | 8.91 | 42.92 | 8.31 | 44.30 | 9.36 |
| Dysregulation | 40.40 | 10.60 | 43.27 | 12.45 | 40.40 | 11.55 |
| Competence | 41.95 | 10.40 | 42.44 | 7.77 | 43.57 | 8.94 |
| Maladaptive | 0.06 | 0.08 | 0.06 | 0.09 | 0.05 | 0.08 |
| Social Relatedness | 1.72 | 0.27 | 1.65 | 0.20 | 1.73 | 0.22 |
| Atypical | 0.24 | 0.17 | 0.25 | 0.19 | 0.27 | 0.23 |

acCMV= Asymptomatic cCMV

sd=standard deviation; B=beta coefficient
